# Geographical Distribution of Patients With Chronic Kidney Disease Receiving Hemodialysis Care in Northwest Iran: A Retrospective Cross‐Sectional Study

**DOI:** 10.1002/hsr2.72545

**Published:** 2026-05-20

**Authors:** Fathiyeh Bahramnejad, Khadijeh Moulaei, Soheil Hashtarkhani, Khadijeh Makhdoomi, Hadi Lotfnezhad Afshar, Bahlol Rahimi

**Affiliations:** ^1^ Department of Medical Informatics, School of Allied Medical Sciences Urmia University of Medical Sciences Urmia Iran; ^2^ Department of Health Information Technology Sirjan School of Medical Sciences Sirjan Iran; ^3^ Spiritual Health Research Center Iran University of Medical Sciences Tehran Iran; ^4^ Department of Pediatrics, Center for Biomedical Informatics University of Tennessee Health Science Center, College of Medicine Memphis Tennessee USA; ^5^ Nephrology and Kidney Transplant Research Center, Clinical Research Institute Urmia University of Medical Sciences Urmia Iran; ^6^ Department of Health Information Technology, School of Allied Medical Sciences Urmia University of Medical Sciences Urmia Iran; ^7^ Health and Biomedical Informatics Research Center Urmia University of Medical Sciences Urmia Iran

**Keywords:** chronic kidney disease, geographical information system, GIS, hemodialysis, spatial analysis, spatial autocorrelation

## Abstract

**Background and Aims:**

Managing chronic kidney disease (CKD) care, particularly for patients requiring hemodialysis, presents significant challenges due to the uneven geographical distribution of cases, which complicates equitable resource allocation. Geographic Information Systems (GIS) can help identify spatial patterns and clusters, thereby supporting more efficient healthcare planning. This study aimed to investigate the spatial distribution of CKD cases requiring hemodialysis in West Azerbaijan Province, Iran.

**Methods:**

This retrospective cross‐sectional descriptive study analyzed hemodialysis patient data collected during January–December 2021. Data from 1709 patients undergoing maintenance hemodialysis were obtained from all 19 governmental and 2 private hemodialysis centers operating at the provincial level. As all registered dialysis centers were included, the data set was considered representative of the provincial hemodialysis population. Records with incomplete key variables were excluded from analysis. Spatial prevalence patterns were assessed using spatial autocorrelation techniques, including the global Moran's *I* and local Moran's *I* indices, to identify nonrandom spatial distributions.

**Results:**

The prevalence of hemodialysis in West Azerbaijan Province was 52.33 per 100,000 population. The global Moran's *I* index was 0.2706 (*Z* = 1.82, *p* < 0.10), indicating a clustered spatial pattern with positive spatial autocorrelation. Local Moran's *I* analysis revealed no high–high clusters of hemodialysis prevalence. However, two significant low–low clusters (cold spots) were identified in the cities of Showt and Mako, while a high–low cluster was observed in Qarah Zia ol Din.

**Conclusion:**

The findings highlight spatial inequalities in hemodialysis distribution and underscore the importance of geographically targeted resource allocation and public health strategies to improve CKD care delivery in the region.

## 1 Introduction

Chronic kidney disease (CKD) is a progressive and long‐term condition characterized by structural or functional abnormalities of the kidneys persisting for at least 3 months, often identified by a reduced glomerular filtration rate or markers such as albuminuria [1]. CKD represents a major public health challenge due to its irreversible nature, high morbidity, and substantial contribution to premature mortality worldwide [2]. Recent global estimates indicate that CKD affects a considerable proportion of the adult population worldwide. A comprehensive systematic review and meta‐analysis reported a global CKD prevalence of approximately 13.4% across all stages, with Stages 3–5 accounting for more than 10% of cases [3]. According to the Global Burden of Disease study, CKD affected nearly 700 million individuals globally in 2017, corresponding to a prevalence of 9.1% [4, 5]. More recent projections suggest that CKD will continue to rise and is expected to become one of the top 5 causes of death worldwide by 2040, reflecting its growing health and economic burden [6].

In Iran, CKD poses a similarly serious challenge. Recent evidence suggests that the prevalence of CKD in the general Iranian population exceeds 15%, which is higher than many global estimates [7]. Consistent with global trends, Iran has experienced a steady increase in end‐stage renal disease (ESRD), leading to a growing demand for renal replacement therapies (RRT), particularly hemodialysis (HD) [8, 9]. The Global Atlas of Kidney Health reported a global RRT prevalence of 759 per million population, with particularly rapid growth anticipated in Asian countries, including Iran [10, 11]. Hemodialysis remains the most commonly used treatment modality for ESRD patients worldwide and serves as the primary RRT in Iran [12, 13]. The increasing reliance on hemodialysis places substantial pressure on healthcare systems due to its high costs, need for specialized infrastructure, and dependence on regular patient access to dialysis centers. In Iran, the number of patients receiving dialysis has risen steadily, with approximately 4000 new patients added annually [14, 15]. As of recent national reports, more than half of Iranian patients with ESRD rely on hemodialysis, underscoring the urgent need for efficient planning and equitable distribution of dialysis services [16, 17]. Inadequate access to dialysis care can accelerate disease progression, increase mortality, and exacerbate health inequalities, particularly in regions with geographic, socioeconomic, or infrastructural disparities.

Geographic Information Systems (GIS) provide powerful analytical tools for examining the spatial distribution of diseases and healthcare services. In epidemiological research, GIS enables the identification of spatial patterns, detection of disease clusters, and assessment of geographic inequalities in access to care [18]. Spatial autocorrelation techniques, such as global and local Moran's *I*, are particularly useful for determining whether disease cases are randomly distributed or spatially clustered, offering valuable insights for health service planning and resource allocation [19, 20, 21]. While GIS‐based analyses have been widely applied to infectious diseases and some chronic conditions, their application to CKD especially in low‐ and middle‐income countries remains limited [22, 23].

Previous studies [16, 24, 25] on CKD and hemodialysis have largely focused on national prevalence estimates, clinical outcomes, or patient‐level risk factors, often without incorporating spatial analysis or geographic visualization. Existing research has seldom examined subnational or provincial‐level variations in CKD burden, nor has it systematically assessed spatial clustering of hemodialysis patients in relation to healthcare infrastructure. Consequently, important geographic disparities in disease burden and service accessibility may remain obscured [24].

West Azerbaijan Province represents a particularly important setting for such an analysis. The province is characterized by a heterogeneous population distribution, border locations, and notable socioeconomic and geographic disparities, all of which may influence access to specialized healthcare services such as hemodialysis. Moreover, the growing burden of chronic diseases, including diabetes and hypertension, suggests an increasing risk of CKD and ESRD in this region. Despite these concerns, spatial evidence on the distribution of hemodialysis patients in West Azerbaijan Province is currently lacking. Therefore, this study aims to analyze the spatial distribution and clustering patterns of CKD patients undergoing hemodialysis in West Azerbaijan Province, Iran, using GIS‐based spatial autocorrelation methods. By providing province‐level spatial evidence, this study contributes novel insights beyond existing national‐level analyses and offers actionable information to support targeted resource allocation, equitable access to dialysis services, and evidence‐based health planning in a developing‐country context.

## 2 Methods

### 2.1 Study Setting

**Figure 1:**
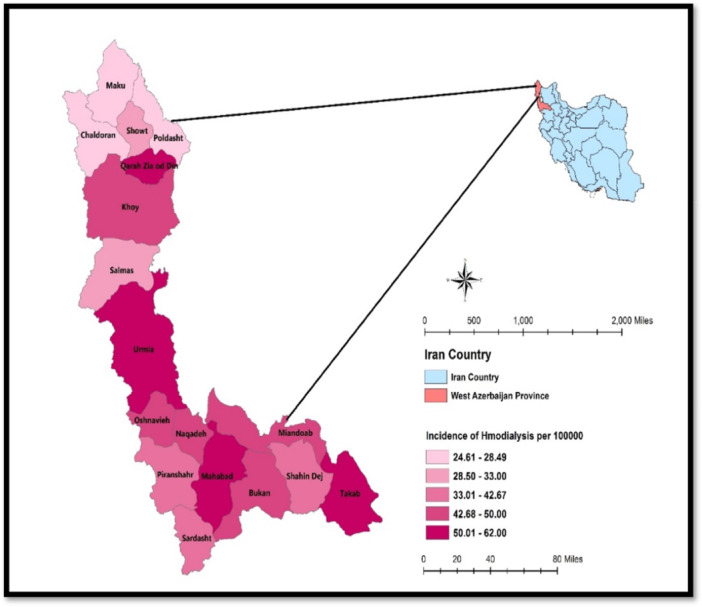
Geographical distribution of CKD prevalence requiring hemodialysis per 100,000 people in West Azerbaijan Province, Iran.

West Azerbaijan Province, 1 of Iran's 31 provinces, is situated in the northwest of the country (Figure 1). Covering an area of 37,312 km^2^, it had a population of 3,265,219 according to the 2016 census, comprising 4.08% of Iran's total population. This makes West Azerbaijan the eighth most populous province in Iran. The population distribution in this province consists of both urban and rural communities, with a significant portion residing in rural areas. This demographic characteristic is an important factor when examining access to health services, including hemodialysis. In this study, a total of 21 hemodialysis centers in the province were examined.

### 2.2 Data Collection

Data were gathered from two primary sources. Hemodialysis patient data, including age, sex, date of birth, number of patients per center, cause of kidney disease, education level, blood type, and weekly hemodialysis sessions, were obtained from the Treatment Deputy of West Azerbaijan University of Medical Sciences. In 2021, 1709 hemodialysis patients were referred to centers in West Azerbaijan Province. Although the data set included pediatric patients and a small number of nonresident individuals treated in the province, only residents of West Azerbaijan Province were included in spatial prevalence calculations and Moran's *I*/LISA analyses. Nonresident patients were retained solely for descriptive analyses.

Variables such as education level and blood type were collected but were mainly used for descriptive purposes and were not included in the spatial accessibility analysis. Population and spatial division data were sourced from the Management and Planning Organization of the province. All spatial analyses were performed using ArcGIS Desktop version 10.7.

### 2.3 Spatial Analysis

**Figure 2:**
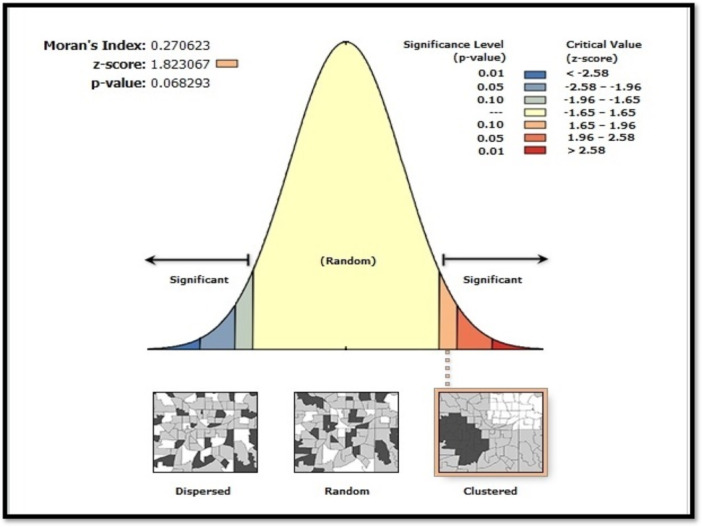
Analysis of the global spatial autocorrelation of CKD prevalence requiring hemodialysis in West Azerbaijan Province, Iran.

The unit of spatial analysis was the city (administrative county level). Hemodialysis prevalence was calculated for each city by dividing the number of resident patients by the city's population per 100,000 people. These city‐level prevalence rates were used as inputs for global Moran's *I* and local Moran's *I* (LISA) analyses. The hemodialysis rate per city was calculated by dividing the number of patients in each city by the population, standardized per 100,000 people. Spatial clustering of hemodialysis cases was analyzed using spatial autocorrelation. Global spatial autocorrelation, measured by Moran's *I*, was used to assess clustering patterns among neighboring regions. Moran's *I*, similar to Pearson's correlation coefficient, provides *p* values, *z*‐scores, and an index value, which indicate significance at the 90%, 95%, or 99% confidence levels. Moran's *I* spatial autocorrelation tool was employed to examine whether the distribution of hemodialysis patients in the study area is clustered, scattered, or random. A positive Moran's *I* value (*I* > 0) suggests spatial clustering, while a negative value (*I* < 0) indicates dispersion. A value close to 0 represents a random distribution (Figure 2).

The analysis utilized seven methods to define spatial relationships, including the inverse distance and Euclidean distance methods. The inverse distance method was chosen, where features closer in proximity have greater influence. A threshold distance of 110 km was selected to ensure that all spatial units had at least one neighbor, minimizing spatial isolation. This threshold was determined empirically and tested in a sensitivity analysis using alternative distances (80, 100, and 130 km), which showed consistent clustering patterns. Hemodialysis rates were standardized per 100,000 population to adjust for population density.

Univariate local Moran's *I* was applied to identify the type and exact location of clusters. To capture regional spatial trends comprehensively, local spatial autocorrelation (LISA) was conducted using Anselin's local Moran's *I*, which helps identify geographic clusters (e.g., hot spots) and evaluates variable relationships across distances. This statistic is valuable for testing spatial variability assumptions and determining the range beyond which spatial relationships cease to exist.

Cluster and outlier analysis, based on Anselin Local Moran's *I*, was performed using ArcGIS software to map clusters and nonclusters. This method identifies hot spots (high–high clusters) and cold spots (low–low clusters), reflecting areas of unusually high or low CKD prevalence requiring hemodialysis, respectively. The analysis also identifies areas where high‐prevalence cities are adjacent to low‐prevalence ones (high–low clusters) and vice versa (low–high clusters). Nonsignificant areas are considered to have no significant spatial association.

### 2.4 Ethical Considerations

This research was approved by the Ethics Committee of Urmia University of Medical Sciences, West Azerbaijan Province, Iran (#IR.UMSU.REC.1398.453). All patient data were anonymized to ensure confidentiality and privacy.

## 3 Results

**Figure 3:**
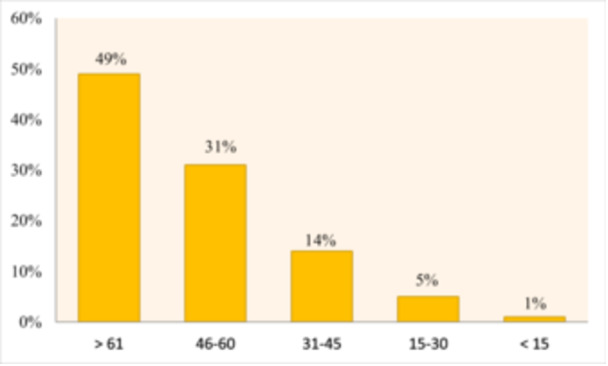
Age distribution of CKD patients requiring hemodialysis.

In 2021, there were 1709 patients undergoing hemodialysis in West Azerbaijan province, resulting in a prevalence rate of 52.33 per 100,000 people. According to statistics from Urmia University of Medical Sciences, 57% of these patients were men, and 43% were women. Individuals over the age of 61 were identified as being at a higher risk for CKD requiring hemodialysis (Figure 3).

**Figure 4:**
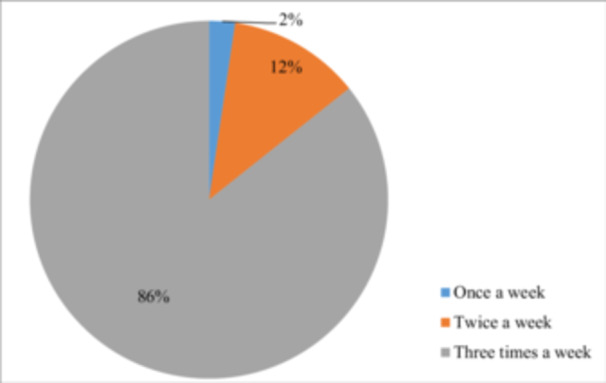
Frequency of weekly hemodialysis sessions among patients.

**Figure 5:**
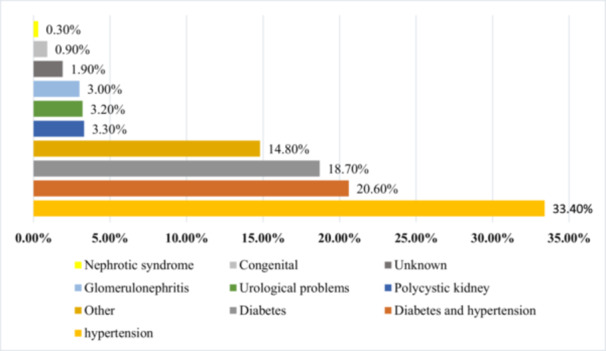
Causes of ESRD in patients.

**Figure 6:**
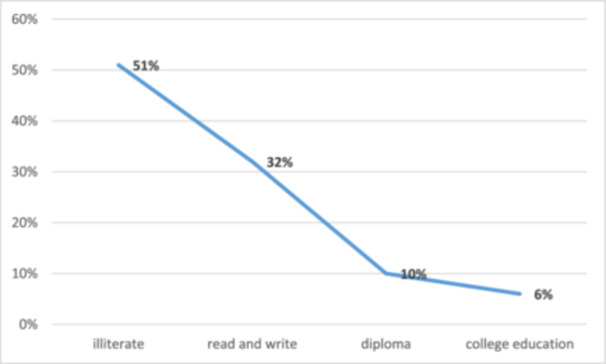
Education level in patients with ESRD.

Figure 4 illustrates the frequency of hemodialysis sessions per week, showing that 86% of patients receive treatment three times a week. The causes of ESRD in this province are depicted in Figure 5, with diabetes and hypertension accounting for 20.6% of cases, and diabetes alone contributing to 18.7%. An analysis of education levels among CKD patients revealed that 51% were illiterate, 32% could read and write, 10% held a diploma, and 6% had a college education (Figure 6).

**Figure 7:**
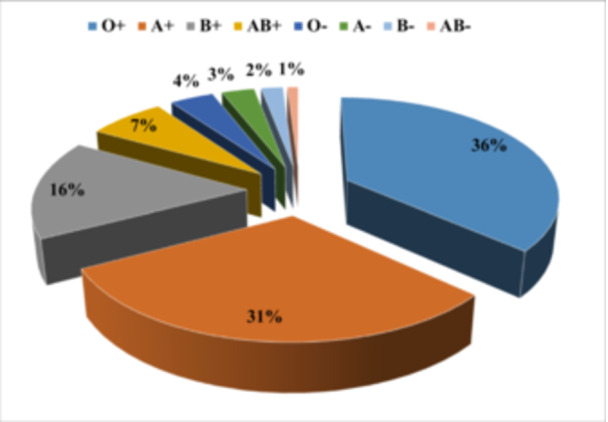
Distribution of blood groups among hemodialysis patients.

Figure 7 presents the distribution of blood groups among hemodialysis patients in the province, indicating that patients with blood group O+ (36%), A+ (31%), and B+ (16%) required hemodialysis the most.

**Figure 8:**
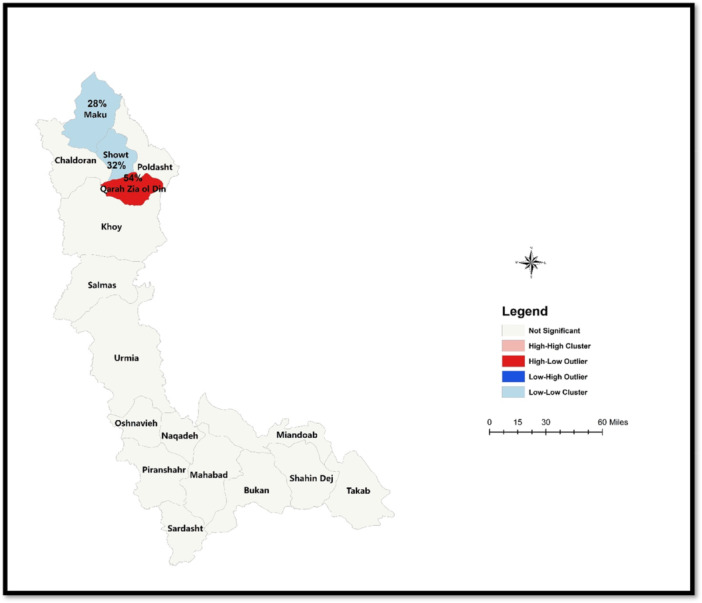
Spatial cluster analysis of CKD prevalence requiring hemodialysis in West Azerbaijan Province.

Spatial cluster analysis of CKD prevalence requiring hemodialysis in West Azerbaijan Province is shown in Figure 8. As explained in Figure 2, spatial autocorrelation was calculated for five different classes. Moran's *I* statistic was used to assess the spatial clustering of hemodialysis cases. The global spatial autocorrelation analysis for 2021 revealed a Moran's index of 0.270623 (*Z* = 1.823067, *p* < 0.10), suggesting a clustered distribution pattern with a positive spatial correlation. The LISA analysis identified both high‐risk and low‐risk clusters. There were no high–high clusters (hot spots) of CKD prevalence requiring hemodialysis in the province, but two significant low–low clusters (cold spots) were observed in Showt and Maku in the northern part of the province. Additionally, a high–low cluster was identified in Qarah Zia ol Din City. The prevalence of CKD requiring hemodialysis in Qarah Zia ol Din, Showt, and Mako was 54%, 32%, and 28%, respectively.

## 4 Discussion

This study investigated the spatial distribution of CKD prevalence requiring hemodialysis in West Azerbaijan Province using global and local spatial analysis techniques. Unlike many previous studies that focused primarily on overall prevalence or individual‐level risk factors, this study specifically aimed to identify geographic clustering patterns and spatial heterogeneity in the distribution of hemodialysis‐dependent CKD. Moran's global statistic provided evidence of a nonrandom, clustered spatial pattern, while Anselin's local Moran's statistic enabled the identification of localized clusters and spatial outliers, particularly in cities where hemodialysis patients are concentrated.

The key finding of this study was the presence of a clustered spatial distribution with positive spatial autocorrelation, in the absence of high–high (hot spot) clusters. Lower prevalence rates observed in cities such as Maku, Chaldoran, and Poldasht may be partly explained by smaller population sizes and cross‐border or cross‐provincial healthcare utilization, rather than a truly lower burden of disease. Local spatial analysis identified two statistically significant low–low (cold spot) clusters in Showt and Maku in the northern part of the province, as well as a high–low spatial outlier in Qarah Zia ol Din City. These spatial patterns represent findings specific to the present study and highlight intraprovincial disparities in the distribution of treated CKD cases requiring hemodialysis. The identified cold spots may indicate more effective prevention or early disease management, but they may also reflect underdiagnosis, limited awareness, or reduced access to diagnostic and dialysis services, emphasizing the need for cautious interpretation and further investigation.

The identified cold spots in Showt and Maku may result from several plausible factors. First, these areas could benefit from more effective preventive measures or early management programs, leading to fewer patients progressing to hemodialysis. Second, lower disease awareness among residents might contribute to underdiagnosis or delayed presentation, resulting in lower observed prevalence. Third, underreporting or incomplete registration in the health information system, especially in remote or border regions, could artificially reduce the recorded number of cases. Additionally, cross‐provincial or cross‐border healthcare utilization may lead patients to seek hemodialysis services outside West Azerbaijan, further contributing to the apparent low prevalence. While these explanations are speculative and require further investigation, acknowledging them is important for interpreting spatial patterns and planning geographically targeted interventions.

Findings from previous studies provide important context for interpreting these spatial patterns. Cerón et al. [26] reported lower ESRD prevalence in Guatemala, which was attributed in part to limited access to diagnostic services rather than a lower true disease burden [26]. This supports our interpretation that low‐prevalence clusters observed in this study may reflect healthcare access limitations rather than reduced CKD occurrence. Sansuk and Sornlorm [27] similarly emphasized the role of socioeconomic factors and access to health services in shaping CKD prevalence patterns in Thailand. Feng and colleagues demonstrated increasing geographic disparities in CKD prevalence across the United States, particularly in southeastern states with higher comorbidity burdens [28]. Liu and colleagues further highlighted the growing global burden of CKD linked to hypertension and emphasized the importance of accounting for regional disparities in health system planning [29]. Bilgel [30] showed that spatial inequalities in ESRD prevalence in the United States are strongly influenced by regional socioeconomic conditions and risk factor distributions. While these studies were conducted in different settings, they reinforce the relevance of spatial analysis for understanding geographic disparities observed in the present study. Urban–rural differences may also contribute to the spatial patterns identified. Oviasu et al. [31] reported higher numbers of diagnosed CKD patients in urban areas, likely reflecting greater disease awareness and access to healthcare services. Consistent with this observation, spatial outliers identified in our analysis may represent referral concentration toward urban dialysis centers rather than localized excess disease risk.

Several contextual factors may explain the spatial distribution of CKD requiring hemodialysis in West Azerbaijan Province. According to the Treatment Deputy of West Azerbaijan University of Medical Sciences, high blood pressure is the leading cause of CKD in the region. The uneven geographic distribution of hypertension, combined with environmental factors such as high salt content in water sources from Urmia Lake and local dietary habits, may contribute to the spatial heterogeneity observed in this study. Additionally, population aging and the increasing prevalence of diabetes, hypertension, hypertriglyceridemia, and hyperuricemia likely influenced by lifestyle factors, metabolic syndrome, and mountainous geography may further shape regional CKD patterns. Tsai and colleagues reported strong associations between hypertension, diabetes, and ESRD in mountainous regions [32], and the US Renal Data System has similarly identified diabetes and hypertension as leading causes of ESRD [32]. These findings provide plausible explanations for the spatial patterns identified, although causal inference is beyond the scope of the current analysis.

This study also found that a higher proportion of men received hemodialysis compared to women (57% vs. 43%). Rather than interpreting this solely as a biological difference, our findings suggest potential gender‐based disparities in disease progression, healthcare utilization, or access to dialysis services within the province. Chan et al. [33] reported that CKD prevalence is often higher in men, while ESRD prevalence may be higher in women [33]. Further spatially stratified analyses are needed to determine whether gender disparities in hemodialysis utilization vary across geographic areas in West Azerbaijan Province. Moreover, Educational level emerged as an important contextual factor. This study found that most patients undergoing hemodialysis were illiterate. Chan et al. demonstrated that lower education levels are associated with increased CKD risk [33]. Low health literacy may intersect with geographic and socioeconomic disadvantage, contributing to delayed diagnosis and progression to ESRD in certain areas. Perneger et al. [34] reported a strong association between lower education levels and ESRD, and Bilgel [30] showed that spatial clusters of poverty, income inequality, and low educational attainment are linked to higher ESRD rates. These findings support the interpretation that the spatial clusters identified in this study may reflect underlying social determinants of health rather than purely clinical risk factors [35].

In addition to socioeconomic factors, hypertension, diabetes, glomerulonephritis, and polycystic kidney disease were identified as major contributors to CKD in the province. Many patients undergoing hemodialysis had low incomes and long waiting times for kidney transplantation, leading to prolonged reliance on dialysis. This suggests that geographic proximity to healthcare facilities alone does not ensure access, as socioeconomic barriers may limit service utilization [36], consistent with findings from Kiani et al. [37], who reported patient relocation toward dialysis centers driven by access constraints.

Overall, the findings of this study emphasize that the spatial distribution of hemodialysis‐dependent CKD in West Azerbaijan Province reflects a complex interaction between disease burden, socioeconomic conditions, and healthcare accessibility. By identifying localized clusters and spatial outliers, this study provides evidence to support geographically targeted interventions, improved diagnostic outreach, and equitable allocation of dialysis resources aimed at improving CKD management and outcomes in the region.

### 4.1 Study Limitation

This study has several limitations that should be considered when interpreting the results. First, the cross‐sectional design provides a snapshot of prevalence and spatial patterns at a single point in time (2021), limiting our ability to infer causality or observe trends. Second, the analysis relied on administrative data from dialysis centers, which may be subject to reporting inconsistencies or under‐ascertainment of cases, particularly in remote areas. Third, while GIS analysis identified spatial clusters, it could not fully elucidate the underlying socioeconomic, environmental, or behavioral determinants of these patterns due to data constraints. Fourth, the inclusion of nonresident patients for descriptive purposes, though excluded from spatial calculations, may still influence the overall provincial profile. Future longitudinal studies incorporating individual‐level risk factor data are recommended to better understand the dynamics and drivers of CKD and hemodialysis distribution in the region.

## 5 Conclusion

This study offers valuable insights into the geographical distribution of CKD patients undergoing hemodialysis in West Azerbaijan Province, Iran. The findings reveal significant clustering in the spatial distribution of these patients, with notable low‐risk clusters identified in the northern cities of Showt and Mako. Additionally, the presence of a scattered high‐low cluster in Qarah Zia ol Din highlights regional disparities in CKD prevalence requiring hemodialysis. These spatial patterns indicate a need for targeted healthcare interventions to address the uneven distribution of CKD care, optimize resource allocation, and enhance patient outcomes in the region.

The application of GIS has proven instrumental in identifying these patterns, demonstrating its potential for improving healthcare planning and management in diverse geographical areas. The results of this study underscore the importance of understanding both the spatial and temporal distribution of CKD prevalence requiring dialysis. This information is crucial for guiding primary prevention efforts and ensuring the effective allocation of health resources.

## Author Contributions

**Fathiyeh Bahramnejad:** software, data curation, formal analysis, methodology, conceptualization, investigation, funding acquisition, writing – original draft, writing – review and editing, visualization, validation, project administration, resources. **Khadijeh Moulaei:** writing – review and editing, conceptualization, investigation, funding acquisition, writing – original draft, visualization, validation, methodology, formal analysis. **Soheil Hashtarkhani:** formal analysis, software, writing – review and editing. **Khadijeh Makhdoomi:** supervision. **Hadi Lotfnezhad Afshar:** writing – review and editing. **Bahlol Rahimi:** investigation, conceptualization, funding acquisition, writing – original draft, writing – review and editing, validation, methodology, software, formal analysis, project administration, visualization, resources, supervision, data curation. All authors read and approved the final manuscript.

## Funding

The authors have nothing to report.

## Ethics Statement

This study was conducted in accordance with the Declaration of Helsinki. Ethical approval was obtained from the Ethics Committee of Urmia University of Medical Sciences (#IR.UMSU.REC.1398.453).

## Consent

As this was a retrospective study using anonymized data, the need for individual patient consent was waived by the ethics committee. All data were handled with strict confidentiality.

## Conflicts of Interest

The authors declare no conflicts of interest.

## Transparency Statement

The lead author Bahlol Rahimi affirms that this manuscript is an honest, accurate, and transparent account of the study being reported; that no important aspects of the study have been omitted; and that any discrepancies from the study as planned (and, if relevant, registered) have been explained.

## Data Availability

The data sets used and/or analyzed during the current study are available from the corresponding author upon reasonable request. Data will be provided upon request.
